# Functional genetic variant of *HSD17B12* in the fatty acid biosynthesis pathway predicts the outcome of colorectal cancer

**DOI:** 10.1111/jcmm.16026

**Published:** 2020-10-28

**Authors:** Yu Lin, Yixuan Meng, Jinying Zhang, Ling Ma, Lu Jiang, Yi Zhang, Ming Yuan, Anjing Ren, Weiyou Zhu, Shuwei Li, Yongqian Shu, Mulong Du, Lingjun Zhu

**Affiliations:** ^1^ Department of Oncology The First Affiliated Hospital of Nanjing Medical University Nanjing China; ^2^ Department of Environmental Genomics Jiangsu Key Laboratory of Cancer Biomarkers, Prevention and Treatment Collaborative Innovation Center for Cancer Personalized Medicine Nanjing Medical University Nanjing China; ^3^ Department of Genetic Toxicology The Key Laboratory of Modern Toxicology of Ministry of Education Center for Global Health School of Public Health Nanjing Medical University Nanjing China; ^4^ Department of Gastrointestinal Surgery The First Affiliated Hospital of Nanjing Medical University Nanjing China; ^5^ Department of Oncology The Jiangyin People's Hospital Wuxi China; ^6^ Department of Biostatistics, Center for Global Health, School of Public Health Nanjing Medical University Nanjing China; ^7^ Department of Oncology The Sir Run Run Hospital Nanjing Medical University Nanjing China

**Keywords:** colorectal cancer, fatty acid biosynthesis, genetic variants, *HSD17B12*, survival

## Abstract

Fatty acids are involved in the development and progression of colorectal cancer (CRC). However, genetic effects of fatty acid biosynthesis pathway on CRC outcome are unclear. Cox regression model was used to evaluate genetic effects on CRC overall survival (OS) and progression‐free survival (PFS), accompanied by calculating hazard ratios (HRs) and confidence intervals (CIs). Differential expression analysis, expression quantitative trait loci analysis, dual‐luciferase reporter assay and chromatin immunoprecipitation assay were performed to explore the genetically biological mechanism. The rs10838164 C>T in *HSD17B12* was significantly associated with an increased risk of death and progression of CRC (OS, HR = 2.12, 95% CI = 1.40‐3.22, *P* = 4.03 × 10^−4^; PFS, HR = 1.64, 95% CI = 1.11‐2.44, *P* = 1.35 × 10^−2^), of which T allele could increase *HSD17B12* expression (*P* = 1.78 × 10^−11^). Subsequently, the functional experiments indicated that rs10838164 T allele could not only enhance the binding affinity of transcription factor YY1 to *HSD17B12* region harbouring rs10838164 but also promote the transcriptional activity of *HSD17B12*, which was significantly up‐regulated in colorectal tumour tissues. Our findings suggest that genetic variants in fatty acid biosynthesis pathway play an important role in CRC outcome.

## 1 INTRODUCTION

Colorectal cancer (CRC) ranks the fourth in cancer incidence and the second in cancer‐related mortality globally, causing serious harm to human health.^1^ In the past decades, great advancements in diagnosis and treatment strategies have been achieved. However, the incidence and morbidity of CRC remain unfavourable in China.^2^, ^3^ Accumulating evidence suggests that environmental, genetic and epigenetic factors all contribute to the development and progression of CRC.^4^, ^5^ It is well known that although the patients with CRC have the same clinicopathologic characteristics and undergo similar therapeutic regimens, the overall survival (OS) still varies from person to person, indicating that genetic factors play an essential role in the variable survival.^6^

Fatty acids, important sources of energy, promote the proliferation of cancer cells by synthesizing cellular membranes and signalling molecules.^7^ The building blocks of fatty acids are mainly derived from exogenous lipids or de novo fatty acid synthesis. Normal cells usually obtain fatty acids in an exogenous dependent manner. While tumour cells are dependent on fatty acids de novo.^8^ Several studies have confirmed that fatty acids are closely associated with tumour growth, invasion, metastasis and drug resistance.^9^, ^10^ Additionally, previous studies suggested that turbulence of biosynthesis and metabolism of fatty acids are involved in the development and recurrence of cancers,^11^, ^12^ including CRC, lung cancer, and prostate cancer.^13^, ^14^, ^15^

Single‐nucleotide polymorphisms (SNPs) are associated with the risk and survival of cancer.^16^ The correlation between SNPs in fatty acid biosynthesis pathway genes and cancer risk has been reported in CRC,^17^ prostate cancer,^18^ and lung cancer.^19^ Moreover, genetic variants in fatty acid biosynthesis are also linked to cancer survival in malignant melanoma.^20^ However, the relationship between genetic variants in fatty acid biosynthesis pathway genes and CRC survival is still unknown.

In this study, we hypothesized that the functional genetic variants in the fatty acid biosynthesis pathway were associated with CRC survival. Herein, we assessed the genetic effect of SNPs in the fatty acid biosynthesis pathway on CRC survival in a Chinese population and investigated the relevant genetic functions.

## 2 MATERIALS AND METHODS

### 2.1 Study participants

In this study, 344 patients were recruited from the First Affiliated Hospital of Nanjing Medical University and Nanjing First Hospital. All participants were histopathologically diagnosed with CRC and unrelated Han Chinese. Meanwhile, 5‐mL blood samples were collected from each patient. Clinical and survival information was collected from medical records and telephone interviews, respectively. The recruitment and follow‐up of population information were started from September 2010, and the deadline for follow‐up data was April 2, 2016.^21^ For all participants, the OS information was missing in 57 patients. OS as the primary endpoint was calculated from the date of CRC diagnosis to the date of cancer‐related death or the last follow‐up. Progression‐free survival (PFS) as the second endpoint was calculated from the date of CRC diagnosis to the date of primary metastasis. The written informed consent was provided by all participants, and the study was approved by the Institutional Review Board of Nanjing Medical University.

### 2.2 Selection of gene and SNP

Kyoto Encyclopedia of Genes and Genomes (KEGG) (https://www.genome.jp/kegg/pathway.html), UniProt (https://www.uniprot.org/) and PubMed (https://www.ncbi.nlm.nih.gov/pubmed/) were used to screen key genes in the fatty acid biosynthesis pathway. We included 31 candidate key genes for further analysis (Table S1 and Figure S1).

For each candidate gene, chromosomal positions were determined in the UCSC Genome Browser (GRCh 37; https://genome.ucsc.edhifu). SNPs located in these genes and the corresponding 2 kb upstream and downstream regions were extracted for association analysis. First, the genotype data were extracted from the 1000 Genomes Project in the populations of Beijing, China (CHB), and Tokyo, Japan (JPT). Quality control was based on three criteria: (a) minor allele frequency (MAF) ≥0.05; (b) Hardy‐Weinberg equilibrium (HWE) of *P* value ≥0.05; and (c) genotyping call rate >95%. Second, PancanQTL (http://bioinfo.life.hust.edu.cn/PancanQTL/), RegulomeDB (http://regulome.stanford.edu/) and HaploReg (https://pubs.broadinstitute.org/mammals/haploreg/haploreg.php) were used to select potentially functional SNPs. As shown in Table S2, SNPs were retained with the expression quantitative trait loci (eQTL) function in colon adenocarcinoma or rectal adenocarcinoma from PancanQTL and a score of <6 from RegulomeDB. Lastly, tagging SNPs were recognized using pairwise linkage disequilibrium (LD) analysis (*r* ^2^ ≥ 0.8) via the HaploView 4.2 software.

### 2.3 SNP genotyping

The details of genotyping are available in previous studies.^21^, ^22^ Briefly, the genomic DNA was extracted from the peripheral venous blood samples using the Qiagen Blood Kit (Qiagen) according to the manufacturer's instructions. DNA was successfully extracted from the samples collected from 344 patients. Genotyping was performed with Illumina Human Omni Zhong Hua Bead Chips. Genotype analysis was independently conducted by two individuals. We followed a uniform quality control protocol to filter the samples and SNPs.

### 2.4 Gene expression and eQTL analysis

We downloaded mRNA expression data of candidate genes on CRC and normal tissues from The Cancer Genome Atlas (TCGA) database (log_2_ transformed), comprising of 625 CRC tissues and 51 normal tissues, and the Gene Expression Omnibus (GEO) datasets(GSE21510, GSE74602, GSE113513, and GSE87211). The relationship between the expression of candidate genes and survival of CRC was also analysed by extracting the data from TCGA database. The GEPIA (http://gepia.cancer-pku.cn/) online tool,^23^ including TCGA databse, was used to assess the expression pattern of candidate genes in pan‐cancer. The Human Protein Atlas (HPA) database (http://www.proteinatlas.org/) was used to evaluate the protein expression of the candidate genes. To further confirm the genetic effects of significant SNPs on the mRNA expression levels, we obtained the access to download the genotype data from TCGA database and merged them with the corresponding expression of mRNA.

### 2.5 Luciferase reporter assays

The 1000‐bp region surrounding the rs10838164 C or T alleles and *HSD17B12* promoter region were synthesized and inserted into the pGL3‐basic vector (Promega) by two restriction sites (*Kpn*I and *Nhe*I). The synthesized plasmids and Renilla plasmids were co‐transfected into CRC cell lines (DLD‐1 and HT29 were seeded into 24‐well plates with a density of 3 × 10^5^ cells/well) using Lipofectamine 3000 (Invitrogen) according to the manufacturer's protocols. After 24 hours of incubation, Firefly and Renilla luciferase activities were measured using the dual‐luciferase kit (GeneCopoeia). Relative luciferase activity was normalized to the Renilla luciferase.

### 2.6 Transfection

DLD‐1 and HT29 cells were seeded into a six‐well plate at 50% confluence and transfected with specific siRNA targeting *YY1*, *YY1* overexpressed plasmids, and corresponding control plasmids (GenePharma) using Lipofectamine 3000 (Invitrogen). After 48 hours of transfection, we extracted RNA and protein to detect the knockdown or overexpression efficiency.

### 2.7 RNA extraction and quantitative real‐time PCR (RT‐qPCR)

Total RNA was extracted from CRC cells by using TRIzol reagent (Invitrogen). The cDNA was synthesized by using Primescript RT Reagent (Takara), according to the manufacturer's instructions. Quantitative real‐time PCR (RT‐qPCR) was carried out on the StepOnePlus Real‐Time PCR system (Applied Biosystems) with the SYBR Green reagents (Takara). The relative expression was calculated by the 2^−ΔΔCt^ method and normalized to β‐actin. All PCR primer sequences used in this study are listed in Table S3.

### 2.8 Protein isolation and Western blot

Total cellular proteins were isolated by incubating the cells with RIPA buffer containing 1% PMSF (Sigma) on ice for 30 minutes. The protein concentrations were quantified by BCA Protein Assay Kit (Beyotime). Proteins were electrophoresed by 10% SDS‐PAGE and transferred to polyvinylidene fluoride (PVDF) membranes (Millipore). The membranes were then blocked with 5% skimmed milk for 2 hours at room temperature. Subsequently, membranes were incubated with the primary antibodies, including anti‐YY1 (63227, Cell Signaling Technology 1:1000) and anti‐β‐actin (3700, Cell Signaling Technology 1:1000) for overnight at 4°C. Next morning, the membrane was incubated with peroxidase (HRP)‐conjugated secondary antibody (Proteintech, 1:5000) for 1 hour at room temperature. Finally, the intensity of the bands was detected by the chemiluminescence system (Bio‐Rad) and analysed by Image Lab software.

### 2.9 Chromatin immunoprecipitation (ChIP) assay

We performed ChIP experiments in DLD‐1 cells using a CHIP‐IT Express Enzymatic kit (53009, Active motif). The assay was conducted in four steps with a sequence as the following: crosslinking, fragmentation, immunoprecipitation and purification. Briefly, cells were cross‐linked with 1% formaldehyde for 10 minutes. Then, the working stock of enzymatic shearing cocktail was added and incubated at 37°C for 15 minutes to shear chromatin. The sheared chromatin was incubated overnight with protein G magnetic beads and YY1 antibodies (63227, Cell Signaling Technology), or normal rabbit IgG as a negative control.*FAM193B*, a known target gene of *YY1*, was selected as the positive control according to the description of YY1 antibodies (63227, Cell Signaling Technology). DNA was purified using the Chromatin IP DNA Purification kit (58002, Active motif). Finally, RT‐qPCR was performed with specific primers for the YY1 binding site (Table S3).

### 2.10 Statistical analysis

Cox regression analysis, including unconditional univariate and multivariate analyses, was conducted to assess the association of the candidate SNPs and CRC outcomes (OS and PFS). Hazard ratios (HRs) and its corresponding 95% confidence intervals (CIs) were calculated to evaluate the genetic effects. To reduce the possibility of false positive findings, we used the false discovery rate (FDR) approach for multiple comparisons. Student's *t* test was used to compare differences in gene expression between CRC and normal tissues. The Kaplan‐Meier curve method was used to assess the association between survival time and the expression levels of gene mRNA. Statistical analyses were performed with R software (Version 3.2.5), PLINK (Version 1.09) and Review Manager Software (Version 5.3). For all statistical analyses, a two‐sided *P* value <.05 was considered statistically significant.

## 3 RESULTS

### 3.1 Association between SNPs in the fatty acid biosynthesis pathway and CRC survival

**FIGURE 1:**
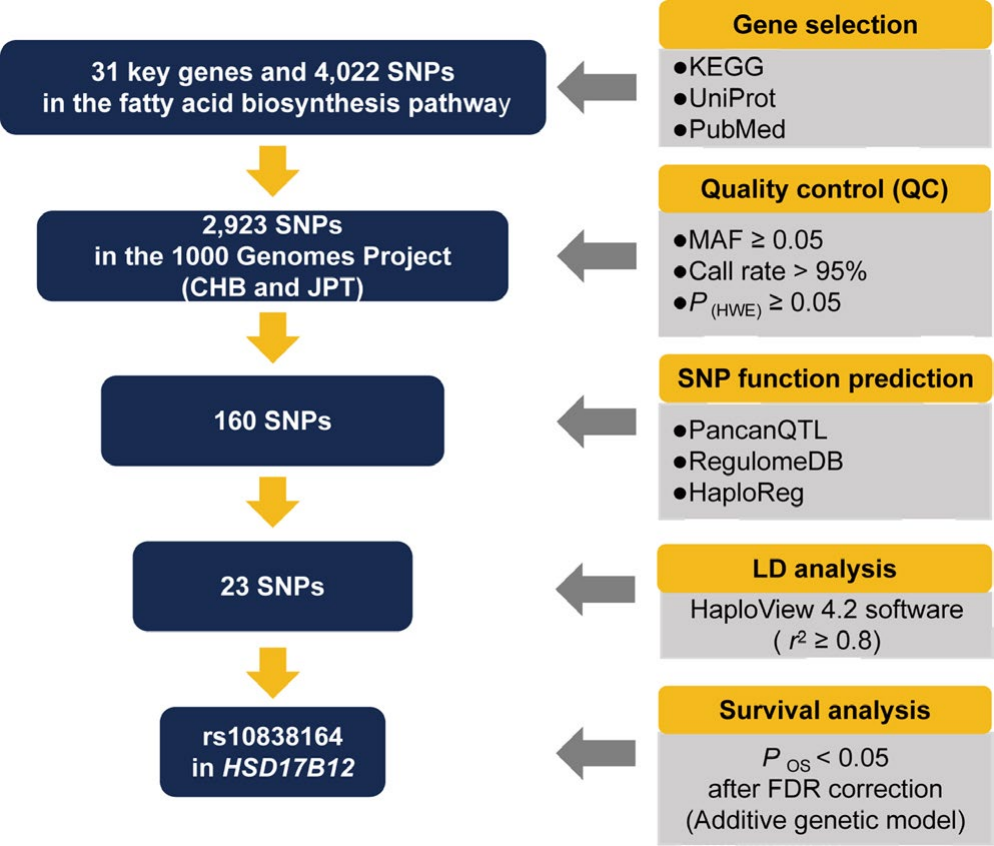
The flow chart of selecting SNPs in fatty acid biosynthesis pathway genes. CHB, Han Chinese in Beijing; JPT, Japanese in Tokyo; MAF, minor allele frequency; HWE, Hardy‐Weinberg Equilibrium; LD, linkage disequilibrium; SNP, single‐nucleotide polymorphism; FDR, false discovery rate; OS, overall survival

As shown in Figure 1, a total of 23 index SNPs in the fatty acid biosynthesis pathway genes were selected for further association analysis after SNP screening and functional annotation. We evaluated the genetic effects of 23 SNPs on the OS of patients with CRC in the additive genetic model (Table S4). Only rs10838164 in *HSD17B12* was found to be associated with OS in patients with CRC after FDR correction (HR = 2.12, 95% CI = 1.40‐3.22, *P = *4.03 × 10^−4^ *, P*_FDR_ = 9.27 × 10^−3^).

**FIGURE 2:**
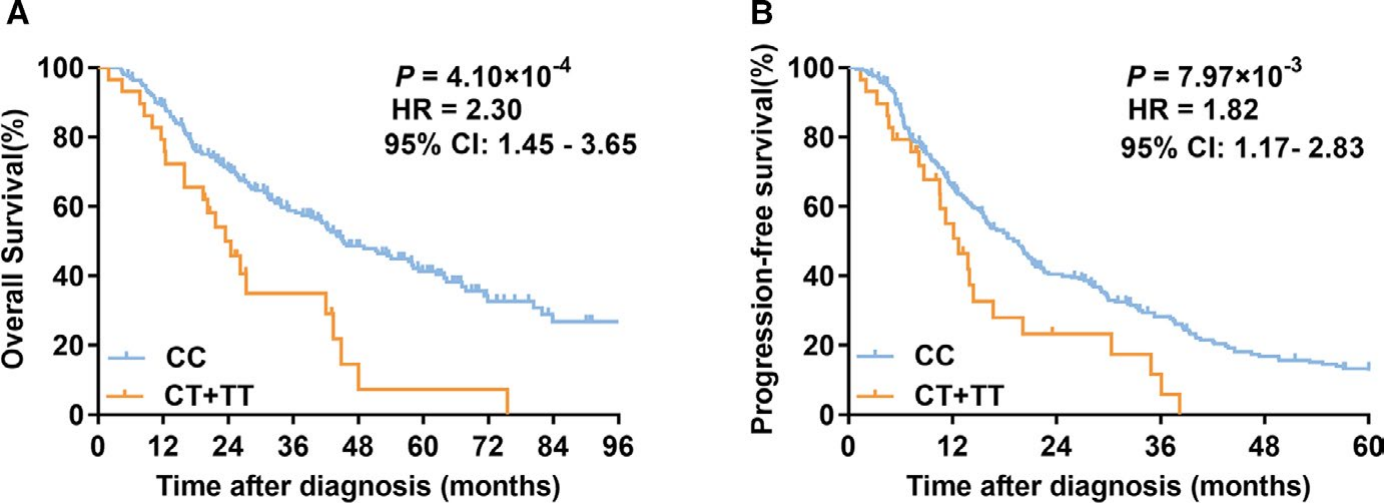
Kaplan‐Meier curves of OS and PFS for rs10838164 in CRC patients. (A) OS; (B) PFS. Patients with incomplete genotype and endpoint information were excluded. Multivariate Cox regression analysis was used to calculate hazard ratios (HRs) and their 95% confidence intervals (CIs), as well as *P* values. OS, overall survival; PFS, progression‐free survival

Next, we dissected the genetic effects of *HSD17B12* rs10838164 on CRC prognosis in different genetic models (Table 1). In the additive genetic model, individuals with T allele were also notably related to an increased risk of progression compared with those with C allele (HR = 1.64, 95% CI = 1.11‐2.44, *P* = 1.35 × 10^−2^ for PFS). A similar genetic effect was found in the dominant genetic model under survival and progression (HR = 2.30, 95% CI = 1.45‐3.65,*P* = 4.10 × 10^−4^ for OS; HR = 1.82, 95% CI = 1.17‐2.83, *P* = 7.97 × 10^−3^ for PFS; Figure 2).

**TABLE 1:** Association of *HSD17B12* rs10838164 with OS and PFS in CRC patients

Endpoints	Genetic models	Genotyping^b^	No. events (%)	Univariate analysis		Multivariate analysis	
				HR (95% CI)	*P*	HR (95% CI)^a^	*P* ^a^
Overall survival	CC	251	131 (43.09)	1.00		1.00	
	CT	28	21 (67.74)	2.29 (1.43‐3.65)	**5.24 × 10^−4^**	2.27 (1.42‐3.63)	**6.37 × 10^−4^**
	TT	1	1 (100.00)	2.57 (0.36‐18.51)	3.48 × 10^−1^	3.29 (0.44‐24.85)	2.49 × 10^−1^
	Missing	7					
	Additive model			2.11 (1.39‐3.20)	**4.13 × 10^−4^**	2.12 (1.40‐3.22)	**4.03 × 10^−4^**
	Dominant model			2.30 (1.45‐3.63)	**3.77 × 10^−4^**	2.30 (1.45‐3.65)	**4.10 × 10^−4^**
	Recessive model			2.32 (0.32‐16.66)	4.02 × 10^−1^	2.92 (0.39‐22.03)	**2.98 × 10^−1^**
Progression‐free survival	CC	289	218 (71.71)	1.00		1.00	
	CT	28	22 (70.97)	1.82 (1.16‐2.83)	**8.61 × 10^−3^**	1.81 (1.15‐2.83)	**1.01 × 10^−2^**
	TT	1	1 (100.00)	1.37 (0.19‐9.76)	7.57 × 10^−1^	2.20 (0.30‐16.37)	4.40 × 10^−1^
	Missing	8					
	Additive model			1.64 (1.11‐2.43)	**1.32 × 10^−2^**	1.64 (1.11‐2.44)	**1.35 × 10^−2^**
	Dominant model			1.79 (1.16‐2.77)	**8.91 × 10^−3^**	1.82 (1.17‐2.83)	**7.97 × 10^−3^**
	Recessive model			1.30 (0.18‐9.27)	7.95 × 10^−1^	2.05 (0.28‐15.16)	4.48 × 10^−1^

Abbreviations: CI confidence interval; CRC, colorectal cancer; HR hazards ratio; OS, overall survival; PFS, progression‐free survival.

^a^ Adjusted for age, sex and Dukes stage in Cox regression model.

^b^ Genotyping for patients with corresponding endpoints (OS or PFS).

### 3.2 Stratified analysis for the association between *HSD17B12* rs10838164 and CRC outcome

**FIGURE 3:**
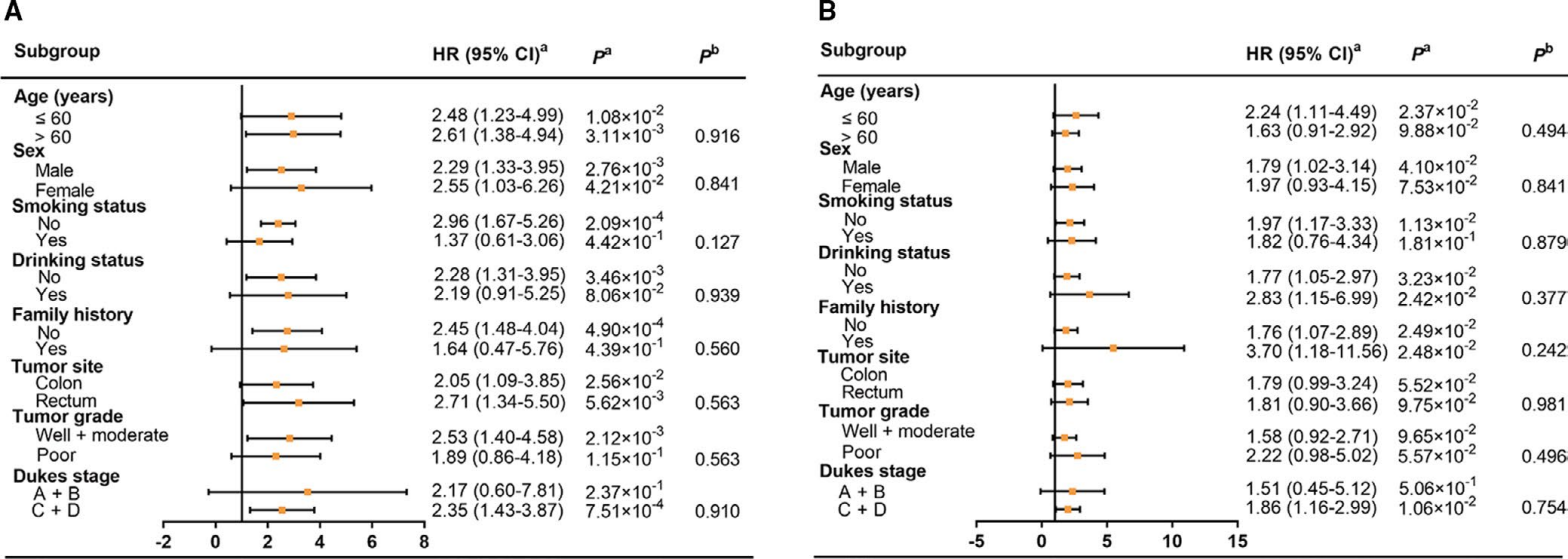
Stratified analysis for the association between *HSD17B12* rs10838164 and outcome of CRC patients in the dominant genetic model. (A) OS; (B) PFS. CI, confidence interval; HR, hazard ratio; OS, overall survival; PFS, progression‐free survival. ^a^Adjusted for age, sex and Dukes stage in Cox regression model. ^b^ *P* value for the heterogeneity

Stratified by demographics (Figure 3A), we found a significant genetic effect of *HSD17B12* rs10838164 on OS in CRC in non‐smokers (HR = 2.96, 95% CI = 1.67‐5.26, *P* = 2.09 × 10^−4^), non‐drinkers (HR = 2.28, 95% CI = 1.31‐3.95, *P* = 3.46 × 10^−3^) and no family history (HR = 2.45, 95% CI = 1.48‐4.04, *P* = 4.90 × 10^−4^). In the subgroup of clinical characteristics (Figure 3A), a significant association was found in the patients with well + moderate (HR = 2.53, 95% CI = 1.40‐4.58, *P* = 2.12 × 10^−3^) and Dukes C + D (HR = 2.35, 95% CI = 1.43‐3.87, *P* = 7.51 × 10^−4^). Additionally, for the association between *HSD17B12* rs10838164 and PFS (Figure 3B), we observed a significant decreased PFS in the patients aged ≤60 years (HR = 2.24, 95% CI = 1.11‐4.49, *P* = 2.37 × 10^−2^), male (HR = 1.79, 95% CI = 1.02‐3.14, *P* = 4.10 × 10^−2^), non‐smokers (HR = 1.97, 95% CI = 1.17‐3.33, *P* = 1.13 × 10^−2^) and the patients with Dukes C + D (HR = 1.86, 95% CI = 1.16‐2.99, *P* = 1.06 × 10^−2^). However, no heterogeneity existed in each subgroup.

### 3.3 SNP rs10838164 allelic‐specific impact on transcriptional activity of *HSD17B12*

**FIGURE 4:**
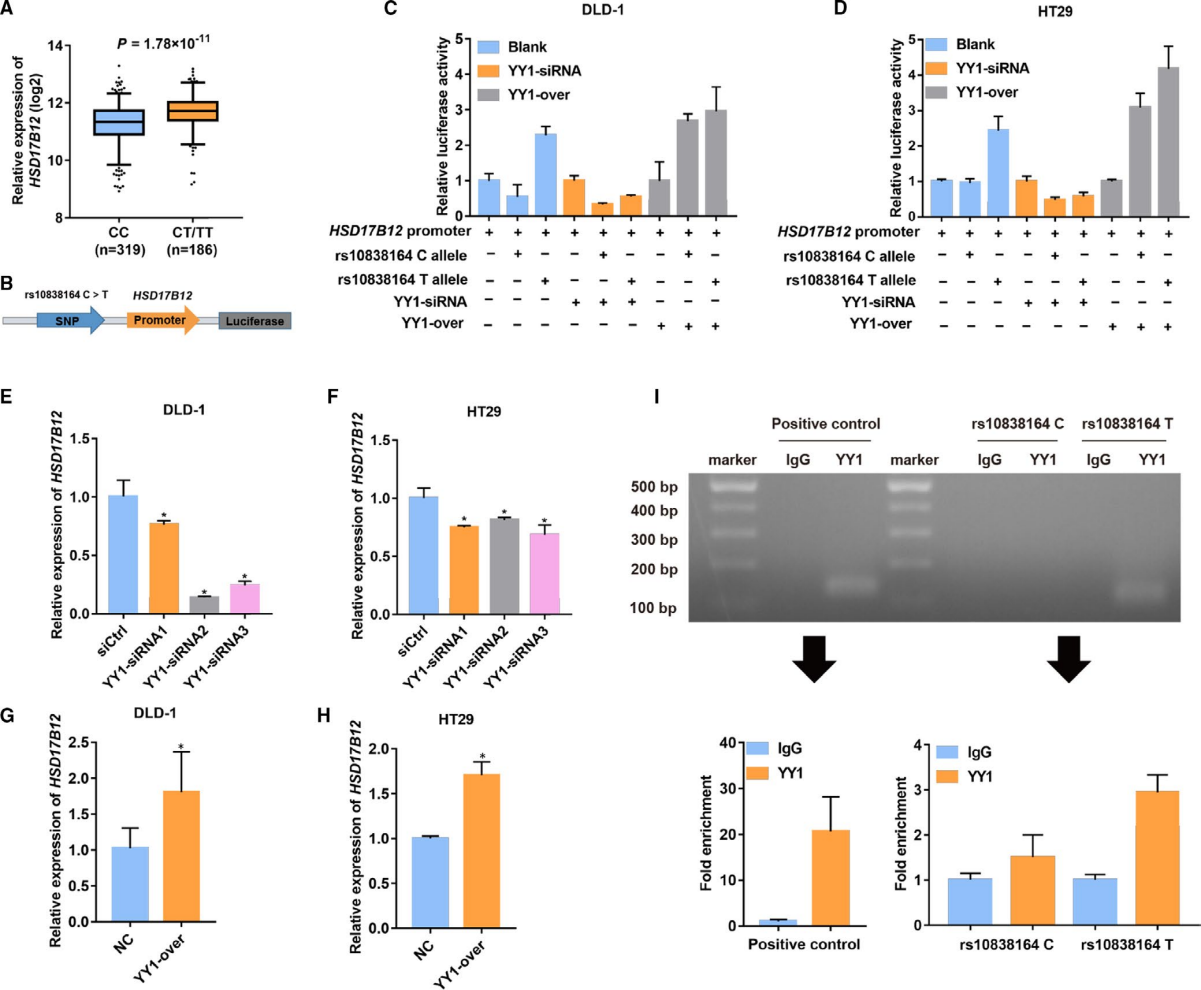
Effect of rs10838164 allele on *HSD17B12*. (A) The eQTL analysis for the genetic effect of rs10838164 on *HSD17B12* expression in colorectal cancer according to the data from the TCGA database; (B) A schematic diagram showed that a region flanking rs10838164 with C or T allele was cloned upstream of the *HSD17B12* promoter‐luciferase reporter vector; (C‐D) *YY1* siRNA or overexpression plasmids were transfected into DLD‐1 and HT29 cells for luciferase activity after 24 h; *HSD17B12* expression was measured using RT‐qPCR in DLD‐1 and HT29 cells after transfection with *YY1* siRNA (E‐F) or overexpression vector (G‐H), *P < .05; (I) ChIP‐qPCR assay was performed to assess the binding affinity between YY1 and the region containing the indicated *HSD17B12* rs10838164 genotype in DLD‐1 cells. The PCR products shown in lanes 1 to 3 were amplified using primers specific for the *FAM193B*, which served as positive controls (lane 1, 100‐ to 500‐bp ladder; lane 2, immunoglobulin G (IgG) binding product as a negative control; lane 3, YY1 binding product), and the products shown in lanes 4 to 8 were amplified with primers specific for rs10838164 alleles (lane 4, 100‐ to 500‐bp ladder; lanes 5 and 6, binding product for rs10808164 C allele, respectively; lanes 7 and 8, binding product for rs10808164 T allele, respectively)

To explore the effect of rs10838164 on *HSD17B12* expression, we conducted eQTL analysis and found that the rs10838164 C>T could significantly increase the expression levels of *HSD17B12* in tumours (*P = *1.78 × 10^−11^; Figure 4A). Considering the notable eQTL and survival effects of rs10838164, we hypothesized that rs10838164 could regulate the expression of *HSD17B12* as a promoter‐like function in CRC. To verify the hypothesis, we conducted luciferase report assays and observed that the luciferase activity of the region containing the rs10838164 T allele was notably higher than that of the region containing C allele in both DLD‐1 and HT29 cell lines (Figure 4B‐D). It suggested that the T allele could enhance the transcriptional activity of *HSD17B12* in CRC.

### 3.4 Effect of rs10838164 on transcriptional activity is mediated by YY1

Previous studies revealed that the SNP in the non‐coding region might regulate gene expression by altering the binding affinity of the transcription factors.^24^ As rs10838164 is located in the intron of *HSD17B12* and based on the above‐mentioned findings, we hypothesized that rs10838164 C>T could enhance the transcriptional activity of *HSD17B12* by affecting the binding affinity of the transcription factor to *HSD17B12*. By bioinformatics analysis (PROMO and JSAPAR), we found that the region surrounding rs10838164 was enriched with transcription factor Yin Yang‐1 (YY1) binding sites, which indicated that rs10838164 T allele might increase *HSD17B12* expression by regulating YY1 binding affinity (Figure S2). To confirm this hypothesis, we transfected *YY1* siRNA or overexpressed plasmids into DLD‐1 and HT29 cells, of which siRNA2 with the highest interference efficiency was selected for further study (Figure S3). Importantly, the different luciferase activity between the rs10838164 C allele and T allele was diminished after the *YY1* deletion, and both alleles could inhibit the activity levels compared with the *HSD17B12* promoter. In contrast, the rs10838164 C allele and T allele both promoted the activity levels after *YY1* overexpression (Figure 4C‐D). Meanwhile, we also performed RT‐qPCR assays to evaluate the *HSD17B12* mRNA expression levels. As expected, knockdown of *YY1* remarkably decreased the *HSD17B12* mRNA expression levels, while overexpression of *YY1* increased the *HSD17B12* mRNA expression levels (Figure 4E‐H). In the TCGA and GEO databases, the expression level of *YY1* also showed a positive correlation with the expression level of *HSD17B12* in CRC cells (Figure S4). Furthermore, we conducted the chromatin immunoprecipitation ChIP assays against a YY1‐specific antibody to assess the binding affinity of YY1 to the region containing *HSD17B12* rs10838164 in DLD‐1 cells. As shown in Figure 4I, our results confirmed that YY1 preferentially bound to the region harbouring T allele using ChIP followed by allele‐specific RT‐qPCR. Taken together, these findings supported that rs10838164 could regulate the expression of *HSD17B12* by affecting the binding of YY1 to *HSD17B12*.

### 3.5 *HSD17B12* expression pattern in CRC tissues

**FIGURE 5:**
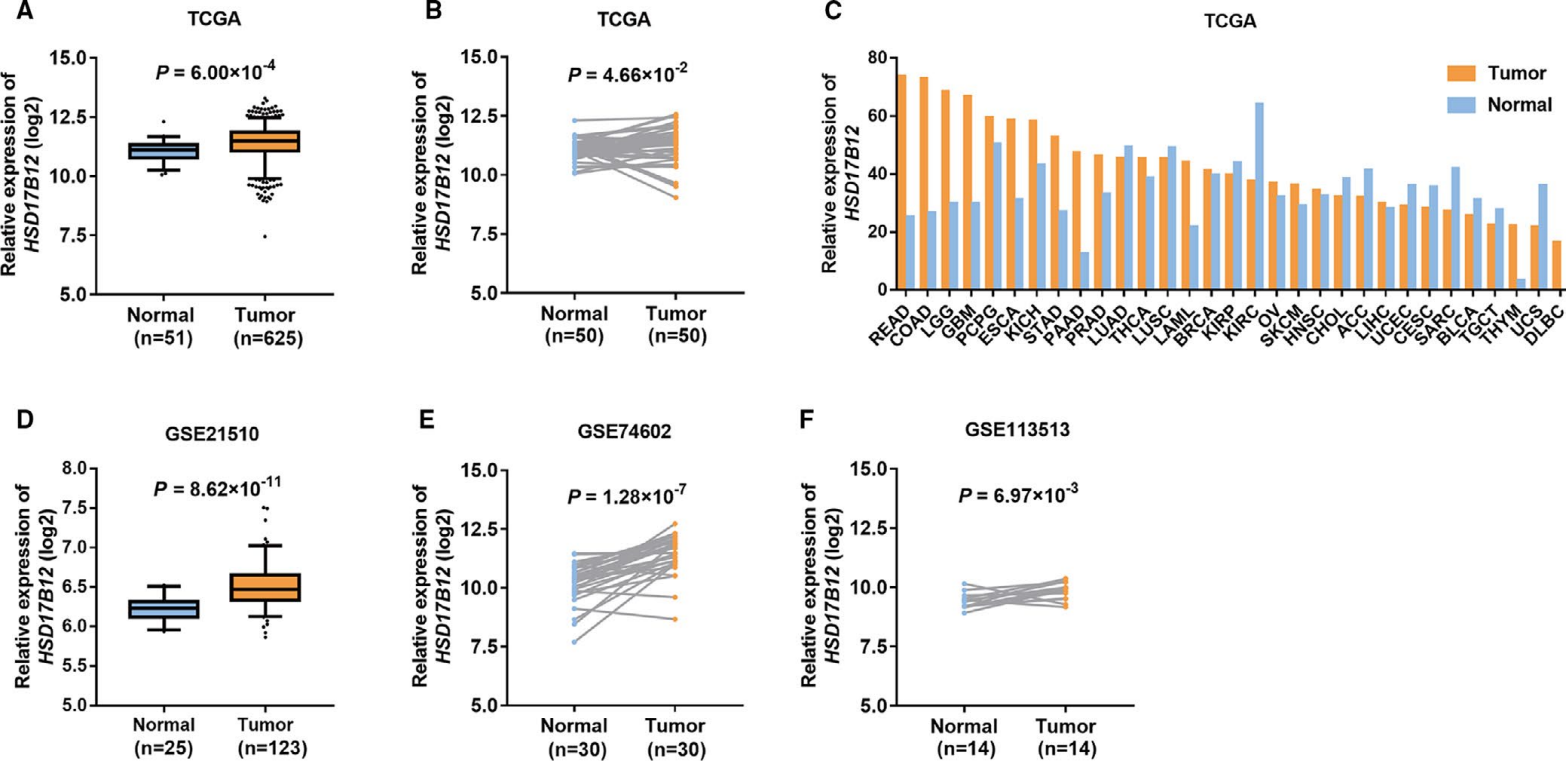
*HSD17B12* expression pattern in CRC tissues. (A) Relative expression of *HSD17B12* was estimated in unpaired colorectal cancer specimens from TCGA database. (B) Relative expression of *HSD17B12* was estimated in paired colorectal cancer specimens from TCGA database. (C) Relative expression of *HSD17B12* was estimated in pan‐cancer specimens from the GEPIA online tool, including TCGA database. The height of bar represents the median expression of *HSD17B12* in colorectal tumour tissues or normal tissues. (D‐F) Relative expression of *HSD17B12* was estimated in colorectal cancer specimens from GEO datasets (D: GSE21510; E: GSE74602; F: GSE113513). Unpaired*t* test or paired*t* test was used for groups comparison. ACC, adrenocortical carcinoma; BLCA, bladder urothelial carcinoma; BRCA, breast invasive carcinoma; CESC, cervical and endocervical cancers; CHOL, cholangiocarcinoma; COAD, colorectal adenocarcinoma; DLBC, lymphoid neoplasm diffuse large B‐cell lymphoma; ESCA, oesophageal carcinoma; GBM, glioblastoma multiforme; HNSC, head and neck squamous cell carcinoma; KICH, kidney chromophobe; KIRC, kidney renal clear cell carcinoma; KIRP, kidney renal papillary cell carcinoma; LAML, acute myeloid leukaemia; LGG, brain lower grade glioma; LIHC, liver hepatocellular; LUAD, lung adenocarcinoma; LUSC, lung squamous cell carcinoma; OV, ovarian serous cystadenocarcinoma; PAAD, pancreatic adenocarcinoma; PCPG, pheochromocytoma and paraganglioma; PRAD, prostate adenocarcinoma; READ, rectum adenocarcinoma; SARC, sarcoma; SKCM, skin cutaneous melanoma; STAD, stomach adenocarcinoma; TGCT, testicular germ cell tumours; THCA, thyroid carcinoma; TNYM, thymoma; UCEC, uterine corpus endometrial carcinoma; UCS, uterine carcinosarcoma

In the TCGA database, *HSD17B12* was highly expressed in colorectal tumour tissues compared with that in normal tissues (*P* = 6.00 × 10^−4^ in unpaired tissues and *P* = 4.66 × 10^−2^ in paired tissues; Figure 5A‐B). In the HPA database, the protein expression of HSD17B12 was also higher in CRC tissues than that in normal tissues (Figure S5). We found that the expression of *HSD17B12* in CRC tissues was the highest among those in the pan‐cancer tissues (Figure 5C). Consistently, this same expression pattern was further validated in GEO datasets (Figure 5D‐F

). Additionally, the expression of the other key genes in fatty acid biosynthesis in TCGA and GEO databases were presented in Figure S6. However, we found no significant association between the expression of *HSD17B12* and OS in patients with CRC (Figure S7).

## 4 DISCUSSION

In this study, we investigated the association between SNPs in the fatty acid biosynthesis pathway and CRC outcome. Our findings suggested that *HSD17B12* rs10838164 was significantly related to the survival of CRC in the Chinese population. SNP rs10838164 showed a gene regulatory role in the expression of *HSD17B1*2 by affecting the binding affinity of YY1 in CRC. Moreover, *HSD17B12* was highly expressed in tumour tissues compared with the adjacent normal tissues from the databases. Therefore, we speculated that rs10838164 might affect the function of *HSD17B12* in CRC by regulating the gene expression levels.

The disorder of metabolism, including the metabolism of fatty acids, has been considered to be one of the important characteristics of malignant tumours, which confers cancer cells the ability to survive, proliferate and metastasize.^25^, ^26^ Previous studies suggested that fatty acid biosynthesis was significantly up‐regulated in tumour cells to provide energy for the synthesis of signalling molecules and membranes.^27^ Additionally, the correlation between fatty acid biosynthesis and tumours has been demonstrated in many aspects, including risk, survival, recurrence and drug resistance.^28^ For example, the expression of *ACLY* is higher in various types of tumour tissues than that in adjacent normal tissues.^29^, ^30^, ^31^ *FASN* is also up‐regulated in cancer tissues and promotes invasion and metastasis of CRC.^32^ Meanwhile, the overexpression of *FASN* is linked to poor survival and drug resistance in breast, prostate and bladder cancers.^33^, ^34^ Furthermore, blocking or deregulating fatty acid biosynthesis can attenuate cancer proliferation, metastasis and recurrence in cancers, including CRC.^35^ All these studies indicate that fatty acid biosynthesis plays a necessary role in tumours.

Recently, the relationship of genetic variants in fatty acid biosynthesis and cancer has been evaluated. For example, genetic polymorphisms in fatty acid biosynthesis are associated with survival in patients with hepatocellular carcinoma and cutaneous melanoma.^20^, ^36^ However, the association between the genetic variant of fatty acid biosynthesis and the survival of patients with CRC has not been well studied.

*HSD17B12*, located at 11p11.2, acts as a multifunctional isoenzyme for the extension of long‐chain fatty acids and the conversion of oestrone to oestradiol, in particular the conversion of palmitic to arachidonic acid.^37^ *HSD17B12* has been identified as an oncogene in various cancers. Studies have shown that *HSD17B12* mRNA expression level is higher in breast tumour tissues than in normal tissues and negatively correlated with prognosis.^38^, ^39^ *HSD17B12* is also gradually up‐regulated with the severity of ovarian cancer.^40^ Moreover, the patients with the overexpression of *HSD17B12* have worse OS and PFS than those with low expression in ovarian cancer.^41^ Consistently, our findings showed that *HSD17B12* was overexpressed in CRC tissues compared with normal tissues. Furthermore, the expression of *HSD17B12* in CRC tissues was the highest among the pan‐cancer tissues, which indicated that *HSD17B12* might play an essential role in CRC. We also found a trend in adverse prognosis between *HSD17B12* expression and CRC although it was not statistically significant. This result may be caused by the analysis that was mostly based on the European population. Therefore, the association between the *HSD17B2* mRNA expression and OS of CRC needs to be explored in the Chinese population.

Genetic variants in *HSD17B12* have been investigated in several cancers. *HSD17B12* rs11037684 A>G is associated with poor OS in patients with cutaneous melanoma.^20^ Genetic variants in *HSD17B12* are associated with a high risk of recurrence in prostate cancer.^42^ Moreover, *HSD17B12* rs7932905 is associated with prostate aggressiveness at the time of diagnosis.^43^ However, no reports have shown the relationship between genetic variants in *HSD17B12* and CRC. Our study is the first to reveal the significant association between *HSD17B12* rs10838164 and outcomes of CRC in the Chinese population. Moreover, stratification analysis showed that rs10838164 T allele predicted worse OS and PFS in patients with Dukes C + D than in patients with Dukes A + B. The eQTL analysis showed that rs10838164 was associated with *HSD17B12* expression and reporter assays indicated that T allele of rs10838164 could increase the expression of *HSD17B12* by altering its transcriptional activity.

Previous studies have shown that the SNP in the non‐coding region might regulate gene expression via altering RNA splicing, RNA degradation and transcription factor binding affinity.^24^, ^44^ The rs10838164 is located in the intron of *HSD17B12*. We observed that the region surrounding rs10838164 was enriched with the binding sites of YY1, which was selected to evaluate the effect of rs10838164 on *HSD17B12* expression. *YY1* is a ubiquitous zinc finger transcription factor that can act as a transcriptional activator or repressor, depending upon the interacting partners.^45^, ^46^ Numerous studies have shown that *YY1* is highly expressed in various tumours and involved in tumour progression by targeting different genes.^47^ As reported by Wang et al,^48^ *YY1* stimulates tumorigenesis and the Warburg effect by up‐regulating *GLUT3* expression in CRC cells. Zhu et al^49^ found that *YY1* activates the *SLC22A15* and *AANAT* expression to promote the proliferation of CRC cells. Furthermore, *YY1* can also suppress fatty acid oxidation by targeting *PGC‐1β* and thus lead to lipid accumulation in liver cancer cells.^50^ We observed that knockdown of *YY1* abrogated the different effects of the rs10838164 C allele and T allele on the transcriptional activity of *HSD17B12*. Additionally, rs10838164 C>T enhanced the binding affinity of YY1 to *HSD17B12*. The *HSD17B12* mRNA expression levels changed in the same direction with *YY1* knockdown or overexpression in CRC cells. Taken together, our findings suggested that rs10838164 C>T could modulate the transcriptional activity of *HSD17B12* by influencing the binding affinity of YY1 and thus altering the *HSD17B12* expression level.

There are several limitations to this study. First, the information about treatments, a factor with the potential effects on CRC survival, is not applicable, which should be adjusted for survival assessment. Second, the number of participants recruited in this study was not large enough to perform stratified analysis in a recessive genetic model. Thus, larger populations are warranted to confirm our findings. Third, we did not validate the relationship between *HSD17B12* expression and SNP rs10838164 in the in‐house clinical samples. Finally, additional functional investigations should be conducted to clarify the function and underlying molecular mechanism of *HSD17B12* on the survival of CRC.

In conclusion, our present study investigated the association between genetic variants in fatty acid biosynthesis genes and CRC survival in the Chinese population. We identified a novel genetic variant, *HSD17B12* rs10838164 C>T, which was significantly linked to worse OS and PFS in patients with CRC. The rs10838164 C>T regulated *HSD17B12* expression by altering the YY1 binding affinity, which might be a possible mechanism affecting the outcome of CRC patients. Moreover, an up‐regulated expression of *HSD17B12* was observed in CRC tissues in both TCGA and GEO databases. Our findings suggest that *HSD17B12* rs10838164 is associated with outcome of CRC, which might be a potential prognostic marker for patients with CRC.

## CONFLICT OF INTEREST

The authors indicate no conflicts of interest.

## AUTHOR CONTRIBUTIONS

**Yu Lin:** Data curation (equal); formal analysis (equal); investigation (lead); validation (equal); visualization (equal); writing‐original draft (equal); writing‐review & editing (equal). **Yixuan Meng:** Data curation (equal); formal analysis (equal); investigation (supporting); validation (equal); visualization (equal); writing‐original draft (equal); writing‐review & editing (equal). **Jinying Zhang:** Data curation (equal); resources (equal); visualization (equal); writing‐original draft (equal); writing‐review & editing (supporting). **Ling Ma:** Formal analysis (supporting); project administration (supporting); supervision (supporting); writing‐review & editing (supporting). **Lu Jiang:** Formal analysis (supporting); methodology (supporting); resources (supporting); writing‐original draft (supporting). **Yi Zhang:** Formal analysis (supporting); resources (supporting). **Ming Yuan:** Resources (supporting); visualization (supporting). **Anjing Ren:** Methodology (supporting); supervision (supporting). **Weiyou Zhu:** Resources (supporting); validation (supporting). **Shuwei Li:** Methodology (supporting); supervision (supporting); writing‐review & editing (supporting). **Yongqian Shu:** Resources (supporting); supervision (supporting). **Mulong Du:** Conceptualization (equal); funding acquisition (supporting); methodology (equal); supervision (equal); writing‐review & editing (equal). **Lingjun Zhu:** Conceptualization (equal); funding acquisition (lead); methodology (equal); project administration (lead); resources (equal); supervision (equal); writing‐review & editing (equal).

## Supporting information

Supplementary Material

## Data Availability

The data are available upon reasonable requests.
