# Supplementary material for: Functional genetic variant of HSD17B12 in the fatty acid biosynthesis pathway predicts the outcome of colorectal cancer
Source: J Cell Mol Med. 2020 Oct 28;24(24):14160–70. doi: 10.1111/jcmm.16026 (PMC7754038; doi:10.1111/jcmm.16026)
Supplement: Supplementary file 1 — Supplementary Material [file JCMM-24-14160-s001.docx]

**Supplementary Table 1. 31 candidate key genes in fatty acid biosynthesis pathway.**

| **Gene** | **Chr** | **Position** | **Definition** | **Functional description** | **PMID** |
| --- | --- | --- | --- | --- | --- |
| *ACACA* | 17 | 35441927-35766902 | Acetyl-CoA carboxylase alpha | Catalyzes the rate-limiting reaction in the biogenesis of long-chain fatty acids. | 20952656 |
| *ACLY* | 17 | 40023179-40075272 | ATP citrate lyase | ACLY is the primary enzyme responsible for the synthesis of cytosolic acetyl-CoA in many tissues. | 23932781 |
| *ACSBG1* | 15 | 78463187-78527049 | Acyl-CoA synthetase bubblegum family member 1 | Mediates activation of long-chain fatty acids for both synthesis of cellular lipids, and degradation via beta-oxidation. | 10954726 |
| *ACSBG2* | 19 | 6135710-6193112 | Acyl-CoA synthetase bubblegum family member 2 | Mediates activation of long-chain fatty acids for both synthesis of cellular lipids, and degradation via beta-oxidation. | 16762313 |
| *ACSL1* | 4 | 185676749-185747215 | Acyl-CoA synthetase long chain family member 1 | Activation of long-chain fatty acids for both synthesis of cellular lipids, and degradation via beta-oxidation. Preferentially uses palmitoleate, oleate and linoleate. | _ |
| *ACSL3* | 2 | 223725732-223808119 | Acyl-CoA synthetase long chain family member 3 | Acyl-CoA synthetases (ACSL) activates long-chain fatty acids for both synthesis of cellular lipids, and degradation via beta-oxidation. ACSL3 mediates hepatic lipogenesis | _ |
| *ACSL5* | 10 | 114135956-114188138 | Acyl-CoA synthetase long chain family member 5 | ACSL5 may activate fatty acids from exogenous sources for the synthesis of triacylglycerol destined for intracellular storage | 17681178 |
| *ACSL6* | 5 | 131285667-131347355 | Acyl-CoA synthetase long chain family member 6 | Activation of long-chain fatty acids for both synthesis of cellular lipids, and degradation via beta-oxidation. | _ |
| *ELOVL1* | 1 | 43829068-43833409 | ELOVL fatty acid elongase 1 | Catalyzes the first and rate-limiting reaction of the four reactions that constitute the long- | 20937905 |
| *ELOVL2* | 6 | 10980993-11044624 | ELOVL fatty acid elongase 2 | Catalyzes the first and rate-limiting reaction of the four reactions that constitute the long- | 20937905 |
| *ELOVL3* | 10 | 103986143-103989344 | ELOVL fatty acid elongase 3 | Catalyzes the first and rate-limiting reaction of the four reactions that constitute the long-chain fatty acids elongation cycle | 20937905 |
| *ELOVL4* | 6 | 80624529-80657315 | ELOVL fatty acid elongase 4 | Catalyzes the first and rate-limiting reaction of the four reactions that constitute the long-chain fatty acids elongation cycle. | 20937905 |
| *ELOVL5* | 6 | 53132196-53213977 | ELOVL fatty acid elongase 5 | Catalyzes the first and rate-limiting reaction of the four reactions that constitute the long-chain fatty acids elongation cycle. | 20937905 |
| *ELOVL6* | 4 | 110970229-111119771 | ELOVL fatty acid elongase 6 | Catalyzes the first and rate-limiting reaction of the four reactions that constitute the long-chain fatty acids elongation cycle. | 20937905 |
| *ELOVL7* | 5 | 60047616-60140101 | ELOVL fatty acid elongase 7 | Catalyzes the first and rate-limiting reaction of the four reactions that constitute the long-chain fatty acids elongation cycle. | 20937905 |
| *FADS1* | 11 | 61567097-61584529 | Fatty acid desaturase 1 | Acts as a front-end fatty acyl-coenzyme A (CoA) desaturase that introduces a cis double bond at carbon 5 located between a preexisting double bond and the carboxyl end of the fatty acyl chain | 10601301 |
| *FADS2* | 11 | 61595713-61634825 | Fatty acid desaturase 2 | Acts as a fatty acyl-coenzyme A (CoA) desaturase that introduces a cis double bond at carbon 6 of the fatty acyl chain. | 12713571 |
| *FADS3* | 11 | 61640998-61659006 | Fatty acid desaturase 3 | This protein is involved in the pathway polyunsaturated fatty acid biosynthesis, which is part of Lipid metabolism | _ |
| *FADS6* | 17 | 72873473-72889705 | Fatty acid desaturase 6 | This protein is involved in the pathway fatty acid metabolism, which is part of Lipid metabolism. | _ |
| *FASN* | 17 | 80036214-80056106 | Fatty acid synthase | Catalyzes the formation of long-chain fatty acids from acetyl-CoA, malonyl-CoA and NADPH. | 7567999 |
| *HACD1* | 10 | 17631958-17659373 | 3-hydroxyacyl-CoA  dehydratase 1 | Catalyzes the third of the four reactions of the long-chain fatty acids elongation cycle. | 18554506 |
| *HACD2* | 3 | 123213363-123303924 | 3-hydroxyacyl-CoA  dehydratase 2 | Catalyzes the third of the four reactions of the long-chain fatty acids elongation cycle. | 18554506 |
| *HACD3* | 15 | 65822827-65870693 | 3-hydroxyacyl-CoA dehydratase 3 | Catalyzes the third of the four reactions of the long-chain fatty acids elongation cycle. | 18554506 |
| *HACD4* | 9 | 21006365-21031635 | 3-hydroxyacyl-CoA dehydratase 4 | Catalyzes the third of the four reactions of the long-chain fatty acids elongation cycle. | 18554506 |
| *HSD17B12* | 11 | 43702143-43878169 | Hydroxysteroid 17-beta dehydrogenase 12 | Catalyzes the second of the four reactions of the long-chain fatty acids elongation cycle | 12482854 |
| *MCAT* | 22 | 43528212-43539403 | Malonyl-CoA-acyl carrier protein transacylase | Catalyzes the transfer of a malonyl moiety from malonyl-CoA to the free thiol group of the phosphopantetheine arm of the mitochondrial ACP protein | 12882974 |
| *OLAH* | 10 | 15085895-15115851 | Oleoyl-ACP hydrolase | Contributes to the release of free fatty acids from fatty acid synthase (FASN). | 26663084 |
| *OXSM* | 3 | 25831563-25836025 | 3-oxoacyl-ACP synthase, mitochondrial | May play a role in the biosynthesis of lipoic acid as well as longer chain fatty acids required for optimal mitochondrial function. | 15668256 |
| *SCD* | 10 | 102106772-102124588 | Acyl-CoA desaturase | Stearyl-CoA desaturase that utilizes O2 and electrons from reduced cytochrome b5 to introduce the first double bond into saturated fatty acyl-CoA substrates | 15907797 |
| *SCD5* | 4 | 83550690-83720010 | Acyl-CoA desaturase5 | Stearyl-CoA desaturase that utilizes O2 and electrons from reduced cytochrome b5 to introduce the first double bond into saturated fatty acyl-CoA substrates. | 15907797 |
| *TECR* | 19 | 14640379-14676792 | Trans-2,3-enoyl-CoA reductase | Catalyzes the last of the four reactions of the long-chain fatty acids elongation cycle. | 12482854 |

**Supplementary Table 2. *In-silico* functional analysis for SNPs annotation.**

| **Chr** | **Gene** | **SNPs** | **Allele^a^** | **Regulome  DB Score** | **PancanQTL^b^** | **HaploReg v4.1** | | | | | | | |
| --- | --- | --- | --- | --- | --- | --- | --- | --- | --- | --- | --- | --- | --- |
|  |  |  |  |  |  | **Promoter histone marks** | **Enhancer histone marks** | **DNAse** | **Proteins bound** | **Motifs changed** | **GRASP QTL hits** | **Selected eQTL hits** | **dbSNP function annotation** |
| 1 | *ELOVL1* | rs839764 | T/A | 4 | Cis-eQTLs | 24 tissues | BRN | 37 tissues | EGR1,ZNF263 |  |  | 30 hits | 5'-UTR |
| 4 | *ACSL1* | rs11132255 | G/A | 5 | Cis-eQTLs |  | 8 tissues |  |  | HP1-site-factor, Sox | 5 hits | 13 hits | intronic |
| 4 | *SCD5* | rs28488833 | G/A | 5 | Cis-eQTLs |  | BRN, GI | BLD |  | Myf |  |  | intronic |
| 4 | *ACSL1* | rs4069938 | T/G | no | Cis-eQTLs |  | LIV | BLD |  | CHD2, TBX5 |  | 16 hits | intronic |
| 4 | *ACSL1* | rs4861631 | A/C | 5 | Cis-eQTLs |  | FAT, BLD, THYM |  |  | Maf, Pou5f1 |  | 6 hits | intronic |
| 5 | *ACSL6* | rs440970 | T/G | 1f | Cis-eQTLs |  | 11 tissues | BLD |  |  | 5 hits | 11 hits | intronic |
| 5 | *ELOVL7* | rs62372102 | A/G | 4 | Cis-eQTLs | 24 tissues | BLD | 11 tissues |  | CTCF, TATA, Znf143 |  | 12 hits | intronic |
| 10 | *ACSL5* | rs10749118 | C/A | 1d | Cis-eQTLs |  |  | 32 tissues | 10 bound proteins | 8 altered motifs |  | 7 hits | intronic |
| 11 | *HSD17B12* | rs10400343 | G/A | 1f | Cis-eQTLs |  |  | HRT,BLD |  | GR, Pou1f1 | 5 hits | 95 hits | intronic |
| 11 | *HSD17B12* | rs10742688 | A/G | 1f | Cis-eQTLs |  |  | HRT,BLD | 5 bound proteins | CCNT2, Zfp691 | 2 hits | 98 hits | intronic |
| 11 | *HSD17B12* | rs10838164 | C/T | no | Cis-eQTLs |  |  |  |  |  |  | 34 hits | intronic |
| 11 | *HSD17B12* | rs12792819 | C/T | no | Cis-eQTLs |  | SKIN |  |  | E4F1, Myc, Spz1 |  | 47 hits | intronic |
| 11 | *FADS2* | rs174575 | C/G | 4 | Cis-eQTLs | 8 tissues | 19 tissues | 32 tissues | 7 bound proteins | NF-E2 | 15 hits | 39 hits | intronic |
| 11 | *HSD17B12* | rs60016711 | C/G | 5 | Cis-eQTLs |  | SKIN | ESDR |  | Pax-5 |  | 37 hits | intronic |
| 11 | *HSD17B12* | rs7118770 | C/T | no | Cis-eQTLs |  |  |  |  |  |  | 55 hits | intronic |
| 11 | *HSD17B12* | rs7942375 | G/A | no | Cis-eQTLs |  |  |  |  | 10 altered motifs |  | 41 hits | intronic |
| 11 | *RP11-613D13.5* | rs939016 | C/G | 5 | Cis-eQTLs |  |  |  |  | 6 altered motifs |  | 71 hits | intronic |
| 11 | *FADS1* | rs99780 | C/T | 2b | Cis-eQTLs | 22 tissues | 4 tissues | 34 tissues | POL2 | 4 altered motifs | 16 hits | 39 hits | intronic |
| 15 | *PTPLAD1* | rs12909321 | C/T | 4 | Cis-eQTLs | 24 tissues | 5 tissues | 32 tissues |  | KAP1, TAL1 |  | 14 hits | 206bp 5' |
| 15 | *PTPLAD1* | rs452306 | C/T | 4 | Cis-eQTLs | 24 tissues | SKIN | 53 tissues | 19 bound proteins | 12 altered motifs |  | 12 hits | 5'-UTR |
| 15 | *PTPLAD1* | rs649121 | T/C | no | Cis-eQTLs |  | BRN |  |  | AP-2, NF-E2, Rad21 |  | 3 hits | intronic |
| 17 | *ACACA* | rs829159 | T/A | 2b | Cis-eQTLs |  |  |  | 4 bound proteins | 5 altered motifs |  | 12 hits | intronic |
| 19 | *ACSBG2* | rs10422296 | T/C | no | Cis-eQTLs |  |  |  |  | 7 altered motifs |  |  | intronic |

^a^Reference/effect allele.

^b^SNPs with function of Cis-eQTLs (SNPs affect local gene expression) in colon adenocarcinoma or rectal adenocarcinoma.

Abbreviations: SNP, single nucleotide polymorphism.

**Supplementary Table 3. List of probes and primers used in this study.**

| **Experiments** | **Description** | **Sequences** |
| --- | --- | --- |
| RT-PCR | *β-actin* | Forward: 5'-AGCGAGCATCCCCCAAAGTT-3' |
|  |  | Reverse: 5'-GGGCACGAAGGCTCATCATT-3' |
|  | *YY1* | Forward: 5'-CCTCTCAGATCCCAAACAACTG-3' |
|  |  | Reverse: 5'-GCCTTTATGAGGGCAAGCTATT-3' |
|  | *HSD17B12* | Forward: 5'-GGTCTTGAAATCGGCATCTTAGT-3' |
|  |  | Reverse: 5'-ATGTTCAGAATAGCCCCTTTGG-3' |
| ChIP | *HSD17B12* (C allele) | Forward: 5'-AGTGAGAATATGCGGTGTT-3' |
|  |  | Reverse: 5'-TGATGAGTTCATGTCCTTCG-3' |
|  | *HSD17B12* (T allele) | Forward: 5'-AGTGAGAATATGCGGTGTT-3' |
|  |  | Reverse:5'-TGATGAGTTCATGTCCTTCA-3' |
|  | *FAM193B* | Forward: 5'-ACTCCCAGTAGACCATAT-3' |
|  |  | Reverse: 5'-ATATTGAGGCAACATAAAGG-3' |
| siRNA | *YY1* siRNA1 | Sense: GACGACUACAUUGAACAAATT |
|  |  | Antisense: UUUGUUCAAUGUAGUCGUCTT |
|  | *YY1* siRNA2 | Sense: GCUAGAAUGAAGCCAAGAATT |
|  |  | Antisense: UUCUUGGCUUCAUUCUAGCTT |
|  | *YY1* siRNA3 | Sense: CCUGAAAUCUCACAUCUUATT |
|  |  | Antisense: UAAGAUGUGAGAUUUCAGGTT |

**Supplementary Table 4. Associations between 23 selected SNPs and CRC survival in additive genetic model.**

| **SNP** | **Allele^a^** | **Gene** | **Location** | **MAF^b^** | **HWE^c^** | **Call rate** | **OS** | | |
| --- | --- | --- | --- | --- | --- | --- | --- | --- | --- |
|  |  |  |  |  |  |  | **HR (95% CI)^d^** | ***P*^d^** | ***P*^e^** |
| rs10400343 | G/A | *HSD17B12* | intronic | 0.245 | 1.000 | 1.000 | 1.15 (0.90-1.49) | 2.69×10^-1^ | 1.00 |
| rs10422296 | T/C | *ACSBG2* | intronic | 0.290 | 0.642 | 1.000 | 1.09 (0.86-1.39) | 4.53×10^-1^ | 7.45×10^-1^ |
| rs10742688 | A/G | *HSD17B12* | intronic | 0.191 | 1.000 | 0.997 | 0.92 (0.69-1.22) | 5.44×10^-1^ | 6.96×10^-1^ |
| rs10749118 | C/A | *ACSL5* | intronic | 0.495 | 0.914 | 0.994 | 0.92 (0.74-1.15) | 4.72×10^-1^ | 6.79×10^-1^ |
| rs10838164 | C/T | *HSD17B12* | intronic | 0.086 | 0.562 | 0.977 | 2.12 (1.40-3.22) | **4.03×10^-4^** | **9.27×10^-3^** |
| rs11132255 | G/A | *ACSL1* | intronic | 0.290 | 1.000 | 0.951 | 1.12 (0.86-1.45) | 4.12×10^-1^ | 8.62×10^-1^ |
| rs12792819 | C/T | *HSD17B12* | intronic | 0.312 | 0.584 | 0.898 | 1.19 (0.92-1.55) | 1.87×10^-1^ | 1.00 |
| rs12909321 | C/T | *PTPLAD1* | 5'-UTR | 0.323 | 0.792 | 0.942 | 1.16 (0.91-1.48) | 2.32×10^-1^ | 1.00 |
| rs174575 | C/G | *FADS2* | intronic | 0.091 | 0.783 | 0.980 | 0.95 (0.66-1.38) | 8.05×10^-1^ | 8.82×10^-1^ |
| rs28488833 | G/A | *SCD5* | intronic | 0.433 | 0.483 | 0.927 | 0.89 (0.70-1.13) | 3.37×10^-1^ | 9.68×10^-1^ |
| rs4069938 | T/G | *ACSL1* | intronic | 0.374 | 0.194 | 1.000 | 0.91 (0.72-1.16) | 4.67×10^-1^ | 7.17×10^-1^ |
| rs440970 | T/G | *ACSL6* | intronic | 0.422 | 0.746 | 1.000 | 0.92 (0.74-1.15) | 4.75×10^-1^ | 6.42×10^-1^ |
| rs452306 | C/T | *PTPLAD1* | 5'-UTR | 0.247 | 0.347 | 0.919 | 1.13 (0.87-1.47) | 3.69×10^-1^ | 8.48×10^-1^ |
| rs4861631 | A/C | *ACSL1* | intronic | 0.089 | 0.249 | 0.991 | 1.04 (0.68-1.58) | 8.73×10^-1^ | 9.12×10^-1^ |
| rs60016711 | C/G | *HSD17B12* | intronic | 0.081 | 0.743 | 0.983 | 0.77 (0.52-1.15) | 1.99×10^-1^ | 1.00 |
| rs62372102 | A/G | *ELOVL7* | intronic | 0.234 | 0.622 | 0.927 | 0.96 (0.72-1.27) | 7.66×10^-1^ | 8.80×10^-1^ |
| rs649121 | T/C | *PTPLAD1* | intronic | 0.059 | 0.436 | 0.930 | 1.60 (0.96-2.66) | 6.93×10^-1^ | 7.97×10^-1^ |
| rs7118770 | C/T | *HSD17B12* | intronic | 0.161 | 0.471 | 1.000 | 0.92 (0.68-1.26) | 6.07×10^-1^ | 7.34×10^-1^ |
| rs7942375 | G/A | *HSD17B12* | intronic | 0.247 | 0.435 | 0.994 | 1.14 (0.86-1.51) | 3.59×10^-1^ | 9.18×10^-1^ |
| rs829159 | T/A | *ACACA* | intronic | 0.231 | 0.388 | 0.997 | 0.99 (0.75-1.30) | 9.18×10^-1^ | 9.18×10^-1^ |
| rs839764 | T/A | *ELOVL1* | 5'-UTR | 0.078 | 0.193 | 1.000 | 1.26 (0.80-1.99) | 3.17×10^-1^ | 1.00 |
| rs939016 | C/G | *RP11-613D13.5* | intronic | 0.108 | 0.596 | 0.983 | 1.16 (0.81-1.66) | 4.19×10^-1^ | 8.04×10^-1^ |
| rs99780 | C/T | *FADS1* | intronic | 0.328 | 0.569 | 0.997 | 0.92 (0.73-1.15) | 4.47×10^-1^ | 7.90×10^-1^ |

^a^Reference/effect allele.

^b^MAF, minor allele frequency in the 1000 Genomes Project (CHB and JPT).

^c^HWE: Hardy Weinberg Equilibrium in the 1000 Genomes Project (CHB and JPT).

^d^Adjusted for age, sex and Dukes stage in Cox regression model.

^e^*P* after FDR correction.

Abbreviations: SNP, single nucleotide polymorphism; HR, hazard ratio; CI, confidence interval; OS, overall survival.

**Supplementary Figure legends**

**Supplementary Figure 1. Key genes in the fatty acid biosynthesis pathway.**

MUFA, monounsaturated fatty acid; PUFA, polyunsaturated fatty acid.

**Supplementary Figure 2. Bioinformatics analysis shows that rs10838164 might affect the binding affinity of transcription factor YY1.** (a) Transcription factor binding sites of intronic SNP rs10838164 was predicted using PROMO. The black arrows indicate the two different alleles of the SNP; (b) The YY1 binding motif was predicted using JASPAR.

**Supplementary Figure 3. The efficiency of *YY1* knockdown and overexpression in CRC cell lines.** The expression of *YY1* was measured by quantitative real-time PCR (a-b) and Western blot (c-d) in DLD-1 and HT29 cells after transfection with *YY1* siRNA or overexpression vector.

**Supplementary Figure 4. The correlation analysis of *YY1* and *HSD17B12* expression in CRC from the TCGA database and GEO dataset.**

**Supplementary Figure 5. Protein expression patterns of HSD17B12 in the CRC tissues and normal tissues from the Human Protein Atlas database.**

(a) Representative immunohistochemical (IHC) images of HSD17B12 protein expression in colorectal normal tissues (left) and cancer tissues (right) in the Human Protein Atlas database. (b) Semiquantitative analysis of the IHC staining intensity of 11 CRC tissues and 3 normal tissues. Two pathologists recalculated the score based on the staining intensity and proportion of positively in a blinded manner. The immunostaining intensity was scored as 0 to 3: 0 (negative staining), 1 (weak staining), 2 (moderate staining) or 3 (strong staining). The proportion of stained cells was scored into four categories: 1 (0-25%), 2 (26-50%), 3 (51-75%) or 4 (76-100%). Multiplication of the intensity and proportion scores was defined as the final score ranging from 0 to 12. Mann-Whitney U test was used to compare the difference of scores.

**Supplementary Figure 6. The 31 key genes expression pattern in CRC tissues**

Relative expression of 31 genes was estimated in unpaired colorectal cancer specimens from (a) TCGA database and (b) GEO (GSE87211). Venn plots showed that 13 of 31 genes were significantly upregulated in CRC tissues in both TCGA and GEO database (c); and three genes downregulated (d).

**Supplementary Figure 7. Kaplan-Meier survival curves for the correlation between *HSD17B12* expression and the OS of CRC patients in TCGA database.** OS, overall survival**.** The median was the cutoff value used to classify *HSD17B12* expression as high or low groups.

**Supplementary Figure 1.**

**
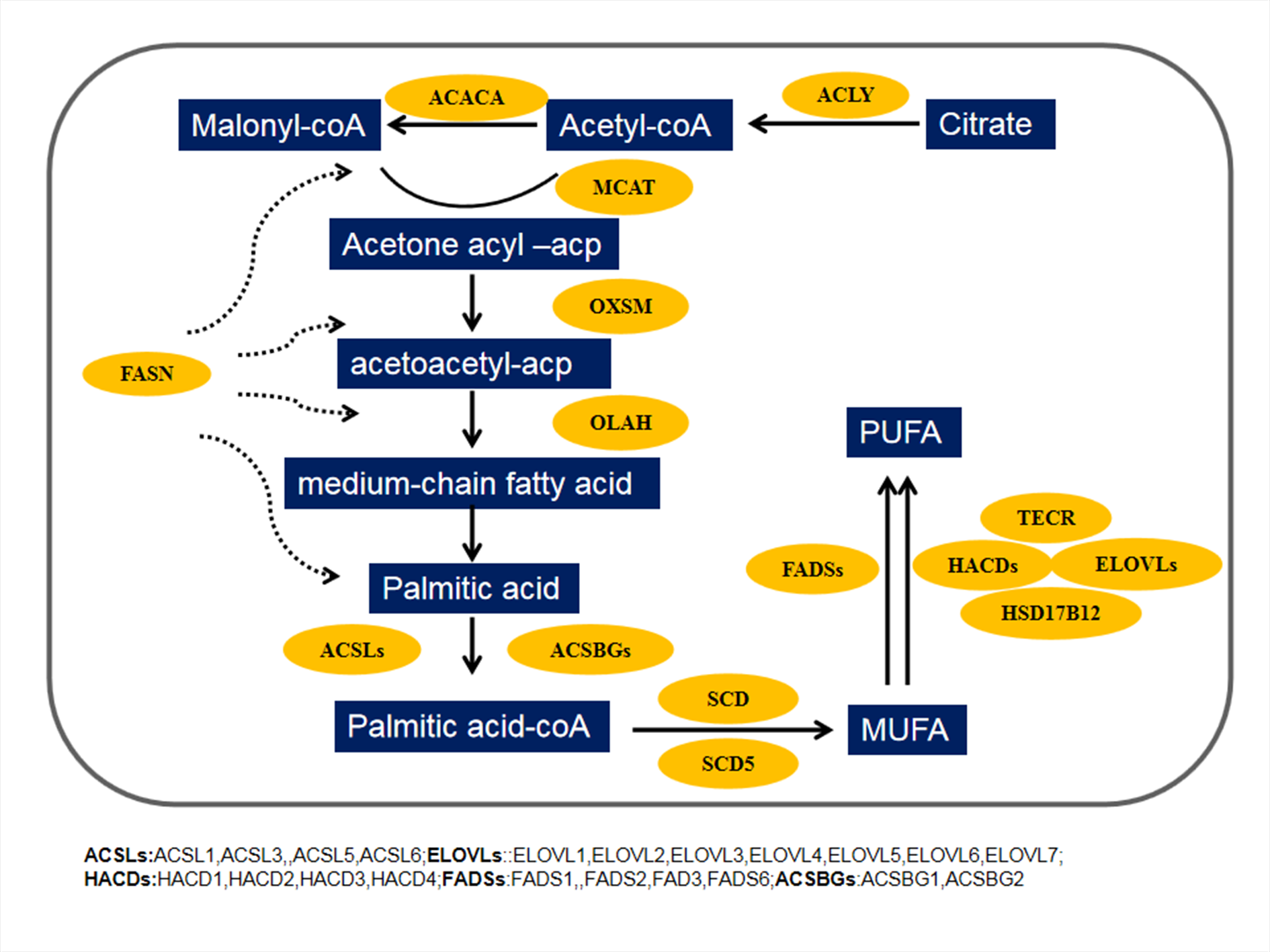
**

**Supplementary Figure 2.**

**
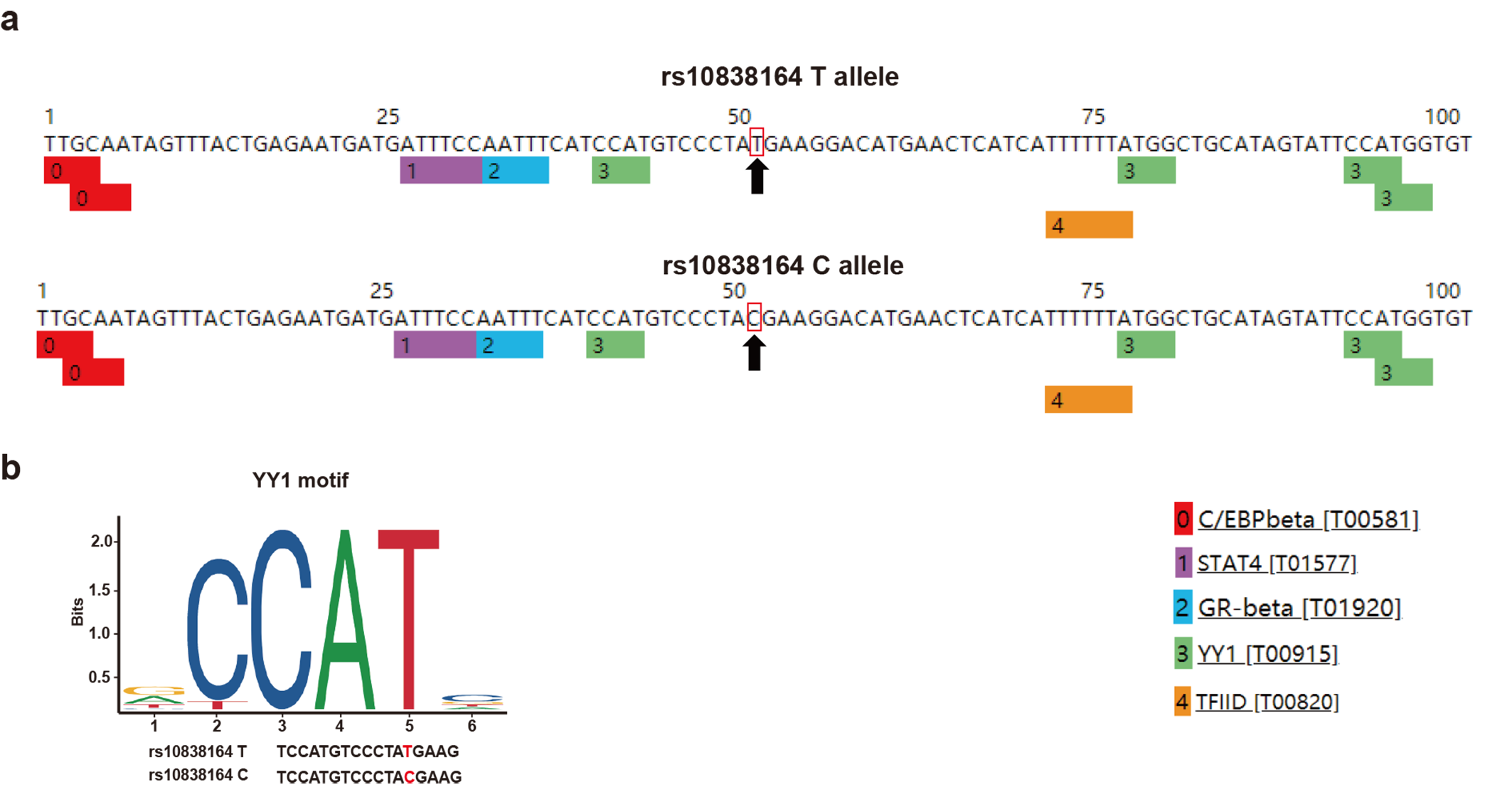
**

**Supplementary Figure 3.**

**
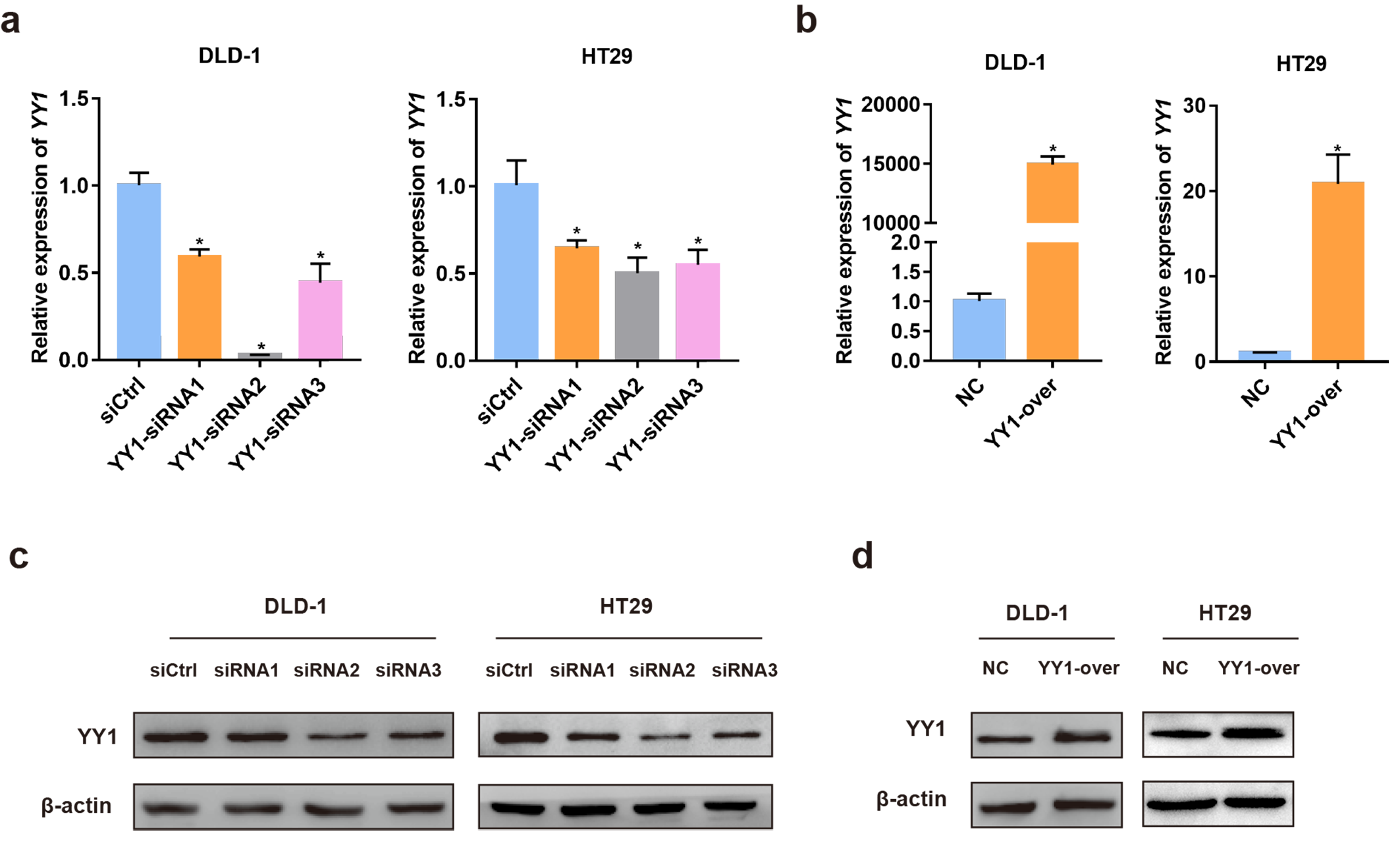
**

**Supplementary Figure 4.**


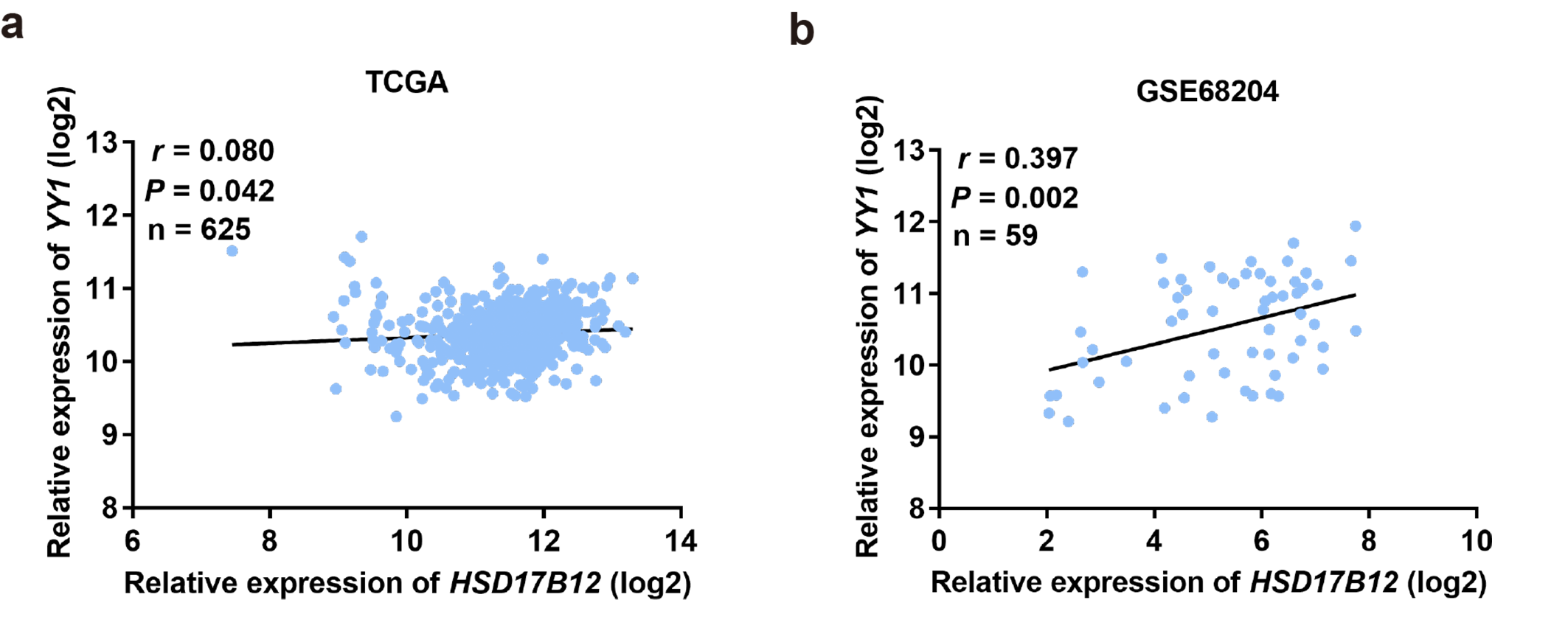


**Supplementary Figure 5.**


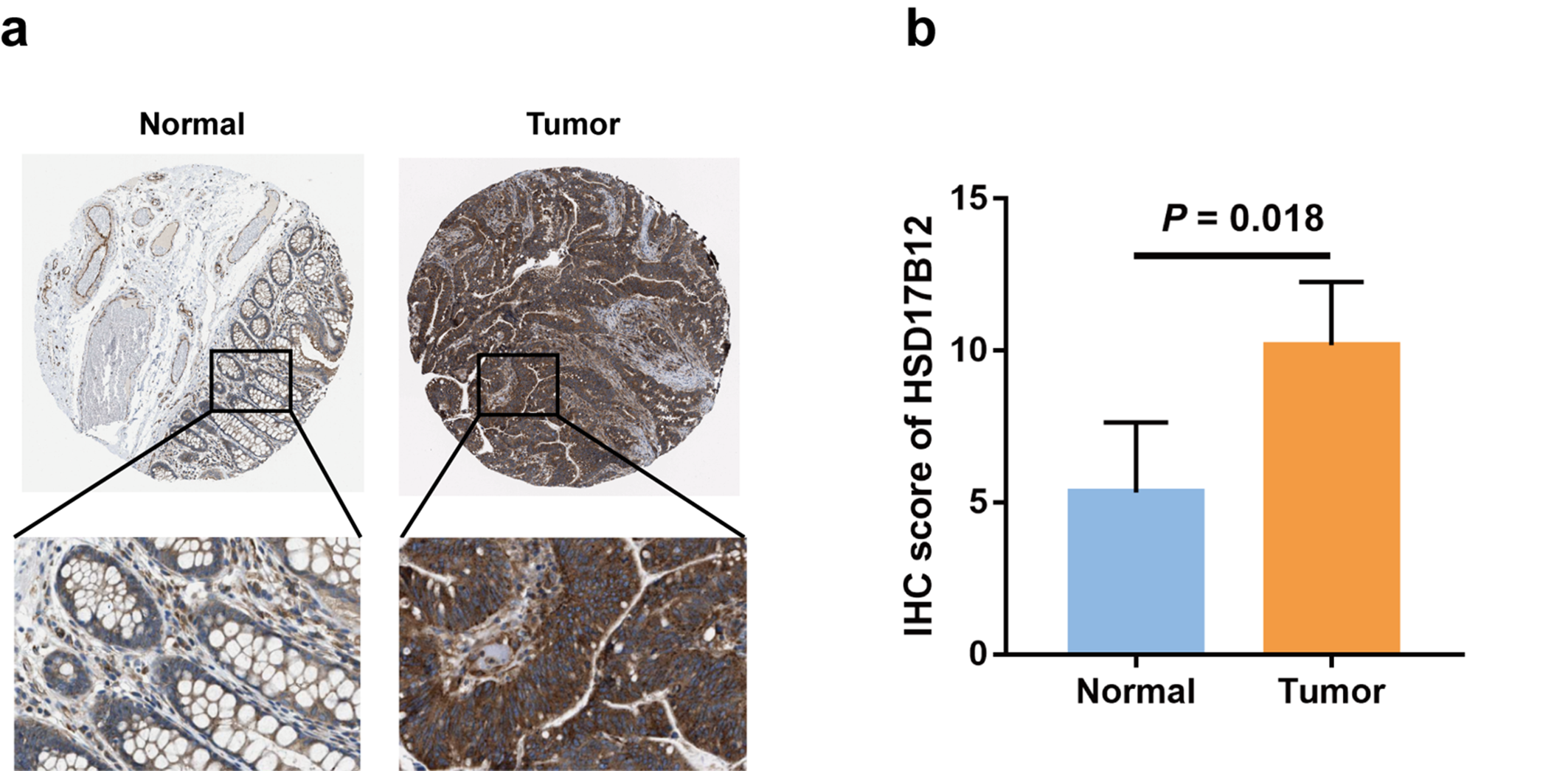


**Supplementary Figure 6.**


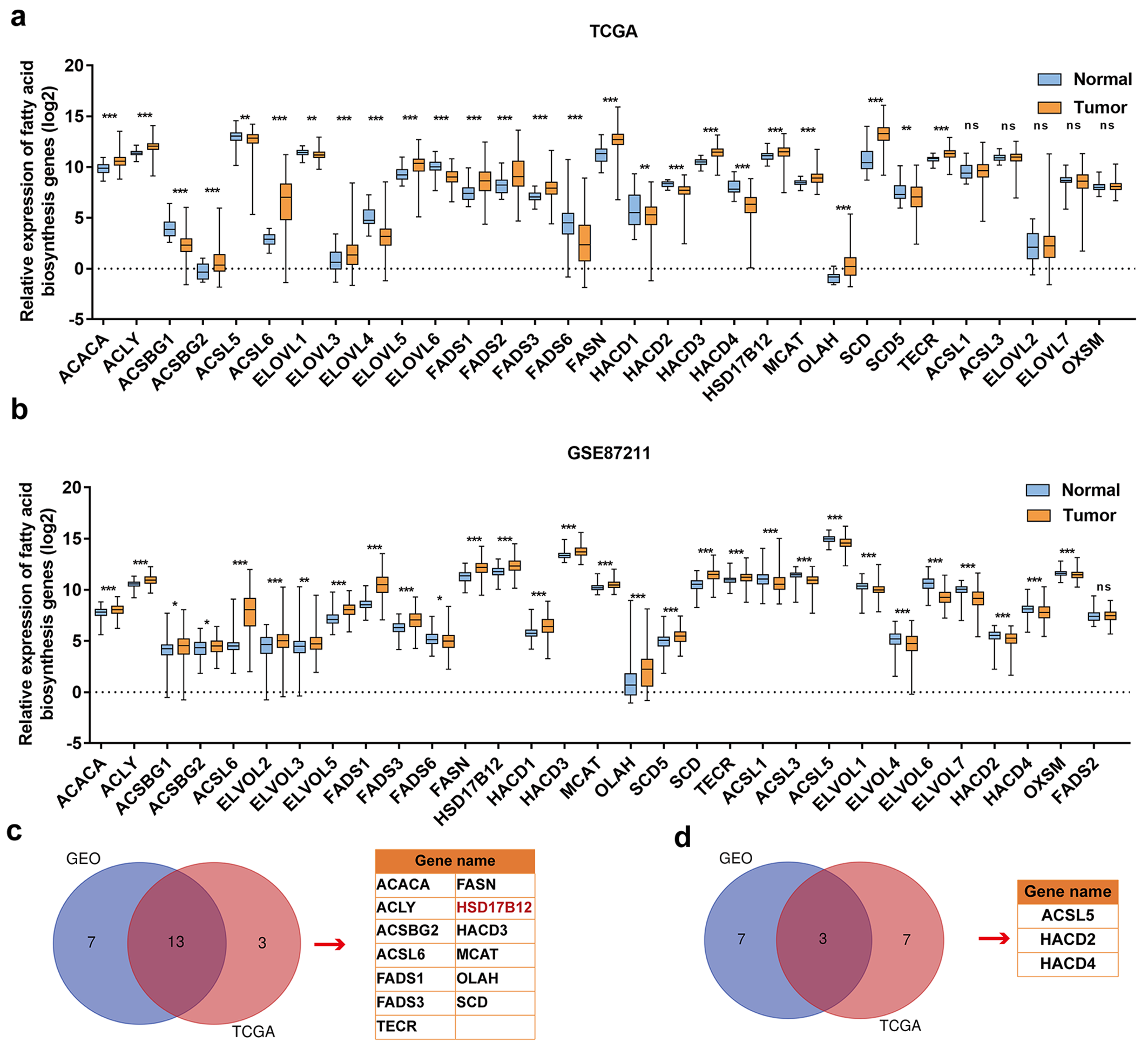


**Supplementary Figure 7.**

**
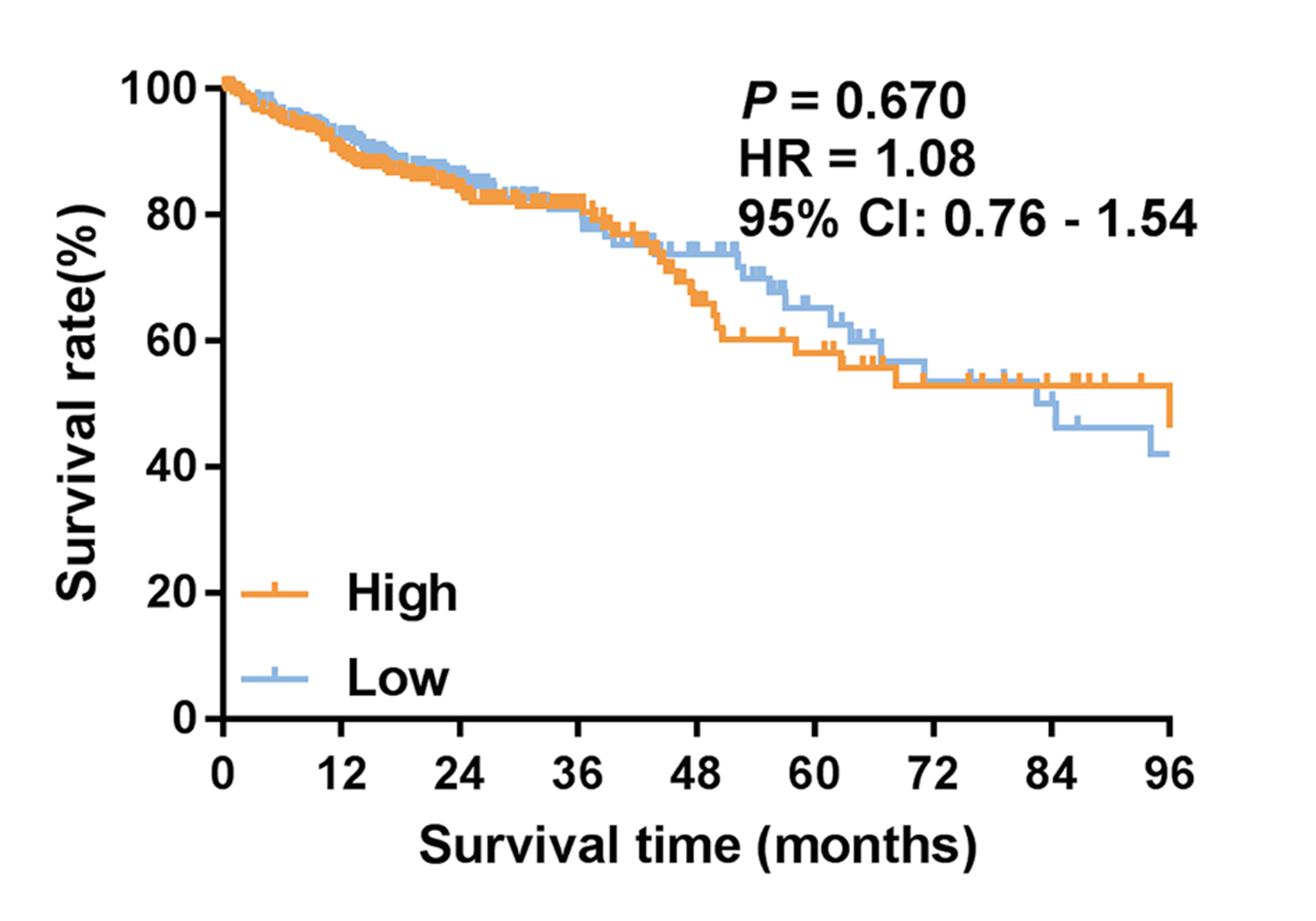
**
